# Identifying adverse childhood experiences and mental health needs among a small sample of pregnant women who use cannabis

**DOI:** 10.3389/fpsyt.2025.1616463

**Published:** 2025-08-25

**Authors:** Aubrey Jones, Lisa M. Blair, Julie A. Kurzer, Kathy Rademacher, Letitia Ducas, Cynthia Cockerham, Kristin Ashford

**Affiliations:** ^1^ College of Social Work, University of Kentucky, Lexington, KY, United States; ^2^ College of Nursing, Wayne State University, Detroit, MI, United States; ^3^ University of Kentucky, Lexington, KY, United States

**Keywords:** cannabis, pregnancy, perinatal mental health, trauma, adverse childhood experiences

## Abstract

**Introduction:**

As the legalization of cannabis becomes more widespread use has steadily increased. Approximately 5 percent of pregnant individuals self-report use during pregnancy.

**Methods:**

This study uses a mixed methods approach to examine adverse childhood experiences, mental health needs, and cannabis use among a small sample (N =59) of women. Cumulative ACE scores of four or more were considerably high within this sample compared to the US average.

**Results:**

Results of a binary logistic regression indicate an association between ACEs and Cannabis use (OR 1.27, 95% CI (1.002- 1.616) when controlling for maternal age and anxiety. Qualitative results identified two main sub-themes: Medical Use for Mental Health Symptoms and Concerns about Negative Mental Health Effects Postpartum.

**Discussion:**

Results suggest a need for patient education about cannabis use in pregnancy and trauma informed care.

## Introduction

Cannabis is the most used psychoactive substance during pregnancy with self-reported use at approximately 5% among all pregnant persons and up to 28% in certain socioeconomically disadvantaged groups (1). The true prevalence of cannabis use in pregnancy is likely higher, given that prior studies have demonstrated that twice as many pregnant persons screen positive for cannabis on a specimen-based drug test than indicate cannabis use by self-report (2). The discrepancy between self-report and specimen-based report of prevalence of cannabis use in pregnancy is unsurprising, considering that there is negative societal stigma and potential prosecution regarding prenatal substance use (3). Nevertheless, due to the changing legal status of non-pregnant adult recreational use of cannabis, the perceptions of risk are decreasing, and cannabis use during pregnancy is increasing (4, 5). Investigation into factors which may contribute to the use of cannabis in pregnancy is warranted to help clinicians identify those at risk for cannabis use and intervene early to promote abstention from or reduction of prenatal cannabis use.

Adverse Childhood Experiences (ACEs) are defined as traumatic events or experiences that occur in an individual’s life before the age of 18 and include experiencing physical abuse, emotional abuse, neglect; witnessing violence within their home; a family member’s death by suicide or growing up in a house where there was substance use, mental health, or justice-involved individuals. ACEs are associated with several negative health outcomes in adulthood, including chronic physical health conditions, poor mental health, and substance use disorders (6). The seminal ACEs study found a significant dose-response relationship between the number of ACEs and risk for negative behavioral, mental, and physical health outcomes and further studies over the decades have corroborated these findings (7–9). Several studies have consistently demonstrated the positive association between ACEs and negative behavioral and mental health outcomes in non-pregnant adults (10, 11). For example, young adults with four or more ACEs were nearly four times more likely to develop a severe Cannabis Use Disorder (CUD) than their peers with no ACES (12). Additionally, it has been reported that non-pregnant adults with four or more ACEs were 3.7 and 4.7 times more likely to develop anxiety and depressive disorders, respectively, than adults with no ACEs (13).

The emerging literature on the intersection of ACEs and prenatal cannabis use has shown a significant positive relationship. It has been reported that pregnant individuals with more cumulative ACEs were more likely to have health conditions which they treated with cannabis than pregnant individuals with fewer ACEs (14). Pregnant individuals with more ACEs have been found to be more likely to test positive for cannabis at either the first prenatal visit or at the time of delivery than pregnant individuals with fewer ACEs (15). It is also known that pregnant individuals who report more ACEs are at greater risk of experiencing both increased substance use and poor mental health than those with fewer ACEs (16). Studies focused on cannabis use must continue to occur as more states continue to legalize cannabis use and stigma associated with use is evolving.

Mental health has been found to be an important factor to consider independently when examining cannabis use during pregnancy. Both anxiety and depressive disorder diagnoses prior to pregnancy increase the odds of cannabis use during pregnancy, by 1.61 and 1.78 times, respectively, compared to those without those mental health diagnoses prior to pregnancy (17). Not only are these preexisting diagnoses associated with increased odds of cannabis use but also increased odds of CUD, with an anxiety diagnosis increasing odds of being diagnosed with CUD by more than three times and a depressive disorder diagnosis increasing odds of being diagnosed with CUD by more than five times in comparison to pregnant people who did not have those diagnoses prior to pregnancy (17). Considering what is known about ACE’s contribution to both anxiety and depression in the general population, it is plausible that ACEs may be influencing the negative mental health of pregnant individuals and their subsequent cannabis use as well.

With increasing legalization, prenatal cannabis use has substantially increased, and cannabis is now the most commonly used federally illegal substance during pregnancy (18, 19). Over the past two decades, the potency of cannabis—particularly tetrahydrocannabinol (THC), its primary psychoactive compound—has nearly tripled (20), raising additional concerns about fetal exposure. Approximately half of pregnant individuals who use cannabis continue to do so throughout pregnancy, with the highest rates of use in the first trimester, a critical period of organogenesis when the fetus is most vulnerable to developmental disruption (19). In addition to increased access, some cannabis dispensaries and online sources actively market cannabis products as a remedy for common pregnancy-related symptoms such as nausea and anxiety, potentially contributing to misperceptions about its safety during pregnancy (21). Despite the perception of cannabis as a natural or safer alternative, prenatal exposure has been linked to lower birth weight (22) and potential neurodevelopmental effects, including attention and behavioral problems in early childhood (23, 24).

The present study examines adverse childhood experiences (ACEs), cannabis use, and mental health needs among a small sample of pregnant women who used cannabis during their current pregnancy. We sought to answer the following questions: 1) Is there a relationship between ACEs and anxiety symptoms among pregnant cannabis users? 2) Does the number of ACEs predict past 30-day cannabis use among pregnant individuals? Data were collected in STATE, a state where cannabis use was not legalized for any purpose at the time of data collection.

## Materials and methods

### Study design

The present study was approved by the University of REDACTED Institutional Review Board (87476-2019). Our study used a convergent parallel mixed-methods design. A survey was designed to collect quantitative data. For the qualitative portion of our study, four focus groups were conducted using a semi-structured interview guide created by the research team. While two mental health-related themes from the focus groups are included here to supplement our quantitative findings, full qualitative results will be reported in a separate manuscript.

### Participant recruitment and data collection

Recruitment efforts occurred from August 2023 through January 2024. Participants were recruited through prenatal clinics at an academic medical center serving urban, suburban, and rural communities in central STATE. The majority of participants were recruited from clinics serving a rural and/or Appalachian population. Eligible participants self-reported cannabis use during their current pregnancy (even if before they knew they were pregnant), were aged 18 to 45, received prenatal care at clinics where recruitment occurred, and were able to read and write in English. Non-English-speaking patients were excluded from participating related to resource constraints at the site level. Potential participants were informed of the study at their prenatal care visit. If participants agreed to learn more, a member of the research team screened patients and informed those eligible about the study during their regularly scheduled prenatal visit. Informed consent and contact information were obtained, after which participants were given a link and asked to complete a cross-sectional survey at their convenience. The survey consisted of 115 total questions, with skip logic to reduce participant burden. Of the 63 individuals who agreed to participate in the study, 60 completed some portion of the survey and 59 completed most measures. A $25 incentive was offered to each participant.

### Qualitative methods

Participants in the survey were randomly invited to participate in focus groups to further explore their perceptions and knowledge of cannabis, prenatal cannabis, and cannabis legalization/policy. Four focus groups (approximately 30 minutes in length) were held via Zoom, with 18 participants. The semi-structured interview guide did not explicitly elicit discussion on ACEs; however, participants were asked open-ended questions about their perceived benefits and concerns of prenatal cannabis use that generated data related to mental health. Each focus group was led by two facilitators, and groups were recorded. Cameras were not required to be on during the group sessions. An additional $25 incentive was provided to focus group participants.

### Demographics and covariates

Participants were asked to provide their age in years (continuous) and to provide their estimated due date to allow calculation of gestational age at survey completion (continuous in weeks). Race was self-reported using the US Census Bureau (2024) five racial categories, with the addition of a category for “more than one race.” Ethnicity was also self-reported, but no participants were Hispanic, thus the variable was dropped. Annual household income was assessed using four categories (<$20,000, $20,000-$49,999, $50,000+, ‘don’t know’). ‘Don’t know’ responses may be due to lack of financial confidence and/or literacy (25) and are thus treated as a valid response using a dummy code rather than as missing data. Marital status was measured using three response options (single, married or living with a partner, divorced or separated). Education was assessed by the highest completed year of schooling (less than high school, GED, high school diploma, some college or trade school, college graduate or above). Employment status was assessed using six categories (unemployed and job seeking, part-time, full-time, student, homemaker, and ‘other’ which included self-employment). Tobacco use was assessed using questions about cigarette smoking and electronic nicotine delivery systems (ENDS), the most common forms of tobacco use in the region, as well as open-ended questions about other types of tobacco or nicotine containing products. Participants were asked the age they first used cigarettes or ENDS (continuous) and whether they used these products in the past 30 days (yes/no).

### Independent variables

The Adverse Childhood Experiences (ACEs) Questionnaire is a measure used to assess childhood trauma (7). The measure consists of ten items regarding personal childhood experiences before the participant’s 18^th^ birthday. Five items consist of personal abuse or neglect and include physical abuse, verbal abuse, sexual abuse, physical neglect, and emotional neglect. The other five items assess family members and parental substance use, mental illness, domestic violence, incarceration, death, abandonment, or divorce. Response options for each question are dichotomous (yes/no). A total score is used and can range from 0 to 10, with higher scores indicating more adverse childhood experiences.

The GAD-7 was used to assess anxiety among participants. This is a 7-item scale that has demonstrated good reliability (*α* = .92). and validity (26). Response options include “not at all,” “several days,” “more than half the days,” and “nearly every day.” Each of the 7-items is scored from 0 to 3. A total score is calculated and ranges from 0 to 21. The GAD-7 has a sensitivity of 92% and specificity of 76% for diagnosis generalized anxiety disorder using a cut-off point of 8 or greater for identifying probable generalized anxiety disorder (27, 28).

### Dependent variables

Cannabis use was assessed by self-report and included questions about age of onset, use in the past 30-days (yes/no).

### Quantitative analysis

Data were analyzed using SAS 9.4. Descriptive statistics (frequencies, means, standard deviations) were generated as appropriate. GAD-7 and ACE measures were scored and summed. Missing data for those individuals who completed the ACE measure (*n* = 57) was minimal for the variables of interest and deemed to be low risk of bias, thus listwise deletion results are reported throughout. Descriptives for demographics and substance use were reported for the full sample (*n* = 59).

The correlation between ACE (summative score) and anxiety (GAD-7 total score) was using Pearson correlation. ACE and cannabis use were then evaluated using binary logistic regression, while controlling for anxiety and maternal age, both of which have been associated with ACE and cannabis use in prior literature. Statistical significance was determined for the logistic model if the overall model *p* value was <.05. For individual predictors, significance was determined by 95% confidence intervals (CI) that do not contain 1.00. Odds ratios are reported for significant predictors.

### Qualitative analysis

Data were transcribed by GMR, a professional transcription company. Two members of the research team reviewed the transcripts compared to the audio recordings for accuracy, and de-identified the transcripts, generated a code book, and coded transcripts to consensus using NVivo software. Additional qualitative analysis and inductive coding was performed independently by a senior author and four research assistants using DeDoose software to examine subthemes. Quotes and subthemes related to mental health were used to contextualize quantitative findings relevant to the primary research question.

## Results

### Demographics

Participants (*n* =59) were on average 26 years old (SD = 4.65, range 18-38), married or partnered (56.6%), employed part- or full-time (58.3%), and had a low income (76.6% with household incomes below $50,000). Most participants had at least some college or trade school (51.7%), though 5% of the sample had not finished high school and 11.7% obtained a GED. Racial make-up of the sample was consistent with the catchment area of the clinics where recruitment occurred, with 85% of participants indicating they were White and non-Hispanic. See **Table 1** for demographics.

**Table 1:** Descriptive statistics.

Variable	Characteristics	*n* (%) or mean [SD]
Age	Maternal age at survey (years) [range: 18–38]	26.39 [4.65]
	Gestational age at survey (weeks) [range: 6–40]	20.47 [11.54]
Maternal race	White	51 (85.0)
	Black or African American	3 (5.0)
	Asian	1 (1.7)
	Native American	1 (1.7)
	More than one race	4 (6.7)
Marital status	Married or living with partner	34 (56.6)
	Single	24 (40.7)
	Divorced or separated	1 (1.7)
Highest completed education	Less than high school	3 (5.0)
	GED	7 (11.7)
	High school diploma	19 (31.7)
	Some college or trade school	25 (41.7)
	College graduate or beyond	6 (10.0)
Annual household income	Less than $20,000	23 (38.3)
	$20,000 - $49,999	23 (38.3)
	$50,000 or more	9 (15)
	Don’t know	5 (8.3)
Employment status	Unemployed and job seeking	10 (16.7)
	Employed part-time	11 (18.3)
	Employed full-time	24 (40.0)
	Student	2 (3.3)
	Homemaker	12 (20.0)
	Other (e.g., self-employed)	1 (1.7)
Tobacco Use	Age at first cigarette (years) [range: 8–25]	15.48 [2.94]
	Smoked 100 cigarettes in lifetime	38 (63.3)
	Smoked cigarettes in past 30 days	22 (36.7)
	Age at first use of ENDS [range: 9–29]	18.34 [4.49]
	Used ENDS in past 30 days	21 (35.0)
Cannabis Use	Age at first use (years) [range: 10–26]	15.58 [3.32]
	Used any cannabis in past 30 days	36 (61.0)
Anxiety	GAD-7 summative score [range: 0–21]	3.61 [2.72]

ENDS, electronic nicotine delivery systems (e.g., e-cigarette).

### Substance use

Participants reported variable rates of tobacco use, with 63.3% reporting that they had smoked at least 100 cigarettes in their lifetime. More than a third of participants (36.7%) had smoked tobacco within the past 30 days, and 35% had used ENDS. These numbers are similar to rates of adult smoking in the region where recruitment occurred. Participants reported initiating cannabis use for the first time at a mean age of 15.58 (SD = 3.32, range 10-26), similar to reported age of initiation of cigarette use (mean 15.48, SD = 2.94, range 8-25). ENDS use was initiated somewhat later (mean 18.34, SD = 4.49, range 9-29). Most participants (61.0%) had continued cannabis use in the past 30 days.

### Adverse childhood experiences

Rates of any reported ACE were high, with 84.2% reporting at least one ACE. Four or more ACEs were reported by 49.1% of the sample. The most frequently endorsed source of ACE was parental separation/divorce (61.4%), followed by emotional abuse (50.9%), physical abuse (40.4%), household member with substance use disorder involving alcohol or ‘street drugs’ (40.4%), and sexual abuse (38.6%). ACEs were positively correlated with anxiety, though this relationship appeared to be relatively weak (Pearson r = 0.29, *p* = .029), answering research question one.

To answer the second research question, binary logistic regression was conducted. The binary logistic regression modeling significantly predicted whether participants endorsed past 30-day cannabis use (*Chi-square* = 8.02, *df* = 3, *p* = .0457). Among the predictors, neither maternal age nor anxiety were significant predictors of past 30-day cannabis use (CI included 1); however, the odds of reporting past 30-day cannabis use rose 27% with each additional ACE (OR 1.27, 95% CI (1.002- 1.616) controlled for maternal age and predictors. See **Table 2** for variable information.

**Table 2:** Adverse Childhood Experiences (ACE).

Variable	*n* (%)	Mean [SD]
Emotional abuse	29 (50.9)	
Physical abuse	23 (40.4)	
Sexual abuse	22 (38.6)	
Witnessed intimate partner violence toward mother or stepmother	12 (21.1)	
Household member with SUD (alcohol, street drugs)	23 (40.4)	
Parents separated or divorced	35 (61.4)	
Household member with depression, mental illness, suicide attempt(s)	23 (21.1)	
Household member went to prison	9 (15.8)	
Frequent emotional neglect	20 (33.3)	
Frequent physical neglect and/or medical neglect	10 (17.5)	
Summative ACE score [range: 0–9]		3.61 [2.72]
Endorsed no ACE	9 (15.8)	
Endorsed 4 or more ACEs	28 (49.1)	

Questions asked about experiences involving parents, stepparents, or adults living in the home. Fifty-seven (*n* = 57) participants answered the questions involving ACEs. SUD, substance use disorder.

### Contextualizing the findings with qualitative data

We sought to garner a deeper understanding of how participants described cannabis in managing mental health symptoms through semi-structured interview guides and focus group participation. Participants in the focus groups did not explicitly identify childhood trauma as a key factor in their discussions, nor were questions in the focus groups geared to elicit such information. Two themes emerged from the focus group data related to mental health: Medical Use for Mental Health Symptoms, and Concerns about Negative Mental Health Effects Postpartum.

#### Medical use for mental health symptoms

In the focus group discussions, individuals commonly identified mental health concerns as among their personal reasons for using cannabis before and during pregnancy. Another identified how not using cannabis during pregnancy has affected her ability to alleviate worry or calm down: “Yeah. And I would say since I did quit, that’s what I miss about it is like being able to calm down or being able to not just be stuck in my mind about like worries and stuff.” Many participants described cannabis use during pregnancy to manage emotional and psychological distress. This included ongoing anxiety, depressive symptoms and stress. One woman shared “There’s definitely been times where treating anxiety was my main reason for use in the first place.” Some participants expressed that cannabis worked better for them than medication: “I do take medicine for my mental health. And I think marijuana helps better than the medicine.”

#### Concerns about negative mental health effects postpartum

Several participants expressed concern about postpartum use related to the experience of paranoia and other mental health effects. One woman who had experience multiple births discussed her experience going back to cannabis postpartum: “The paranoia is definitely real, ‘cause I did it after my second child. With my first child, I was like nope. Can’t do it. I don’t wanna be too paranoid.” Another stated that she had talked to friends about the issue and expressed concern about paranoia, attention, and ‘laziness’:

“I’ve also talked to some of my friends who previously smoked and stopped and had their child. Well, they said that they didn’t jump right back into smoking because they would actually get paranoid. They would think they would hear crying, and the baby was just sleeping. So, that kind of worries me, too, is just, I guess, if I smoke too early, maybe not paying attention or being too paranoid. I know smoking for me made me really lazy. And I don’t wanna be a lazy mom.”

## Discussion

The prevalence of ACEs in this sample was notably high, with 84.2% of participants reporting at least one ACE, and nearly half (49.1%) reporting four or more. Comparatively, 63.9% of U.S. adults reported at least one ACE; 17.3% reported four or more ACEs (29). ACEs were predictive of past 30-day cannabis use in a pregnant sample in a dose-dependent fashion even after controlling for anxiety symptoms and maternal age. This finding is consistent with prior literature which suggests that ACEs are associated with anxiety and other behavioral health concerns (30) and that cannabis is used as a coping mechanism by individuals with trauma exposure (31).

Meanwhile, the qualitative analysis did not identify trauma explicitly as this data was not solicited, yet mental health was a common theme identified in the focus group data. Participants described using cannabis to manage mental health symptoms, particularly anxiety, and shared that cannabis helped them calm down or reduce stress during pregnancy. Multiple participants discussed the calming effects of cannabis or discussed the ways in which in “chilled” them out and made it easier to focus. Although cannabis may offer a temporary solution to managing mental health needs, participants also discussed their concerns about negative mental health effects in the postpartum period describing experiences of paranoia. These results underscore the need for comprehensive mental health support for pregnant and postpartum individuals, particularly for those who use/d cannabis before or during pregnancy.

Given the increased use of cannabis in pregnancy and its risk to the fetus, we recommend providing patient education for all pregnant and postpartum women on the risks associated with cannabis use. Providing options for mental health support including therapy or medication along with patient education regarding the risks and benefits of these options compared to cannabis offers a pragmatic first step. Similarly, identifying trauma history early in pregnancy offers an opportunity for healthcare providers to address physical and mental health needs of patients given the extent literature on ACEs and health outcomes and downstream effects of trauma on health. However, providers must also consider identifying trauma histories of patients can be done in a safe way that reduces the potential harm (Department of Health (32) as cited in 33). When working with individuals who have experienced trauma, taking a trauma informed care (TIC) approach is recommended. TIC has become a prominent approach in mental health professional practice, including social work (34). TIC describes an approach to responding to trauma in human service settings and acknowledges that organizations, service providers, and service recipients experience collective trauma (35) and, seeks to improve outcomes by acknowledging trauma responses and centering the humanity of those who have experienced trauma (36) by resisting pathologizing trauma responses (37). Moreover, trauma-informed approaches recognize that the experiences of trauma are widespread across all demographics and have an impact on all people engaged in a system, including patients, staff, and families (38). These early approaches have the potential to prevent or identify anxiety and mental health challenges early on in pregnancy, allowing patients to connect with resources that promote healthy coping and address underlying causes of cannabis use. Trauma informed care requires a systemic change in how trauma is identified and addressed (38).

### Limitations

We collected data at one healthcare system in STATE from 59 women for this small pilot study. This limited the generalizability of findings. Additional research is needed with a larger pool of participants and the inclusion of a wider cohort of individuals across racial and ethnic groups. This is particularly true given the more widespread use of cannabis among racial and ethnic minority groups compared to White women particularly in rural areas and the Appalachian region (39). Due to the self-selection nature of our study for both surveys and focus group participation, self-selection bias likely affects this convenience sample. Social desirability bias and fear of legal repercussions and judgment stemming from disclosure of use may have prevented eligible participants from requesting information about the study. Similar concerns may arise related to the self-report measure of past 30-day use. Lastly, the ACE measure focuses on childhood trauma and does not address potential ongoing trauma which may influence cannabis use. Future research should build on this study and include larger samples of pregnant women who use cannabis, longitudinal examinations of effects across the full perinatal period, and biochemical confirmation of self-reported abstinence and use.

## Conclusion

Our study examined the intersection of adverse childhood experiences (ACEs), cannabis use, and mental health outcomes among a small sample of pregnant women who used cannabis during their current pregnancy. Consistent with prior literature, we discovered that for every increase in ACE scores, the odds of reported cannabis use increased. Focus group data revealed reasons for cannabis use in pregnancy such as managing mental health symptoms while also revealing participants concerns about future mental health symptoms in the postpartum period. Comprehensive mental health initiatives are needed to support pregnant individuals with histories of trauma and cannabis use.

## Data Availability

The raw data supporting the conclusions of this article will be made available by the authors, without undue reservation.
